# Nanoscale mechanisms in age-related hip-fractures

**DOI:** 10.1038/s41598-020-69783-5

**Published:** 2020-08-26

**Authors:** Shaocheng Ma, En Lin Goh, Tabitha Tay, Crispin C. Wiles, Oliver Boughton, John H. Churchwell, Yong Wu, Angelo Karunaratne, Rajarshi Bhattacharya, Nick Terrill, Justin P. Cobb, Ulrich Hansen, Richard L. Abel

**Affiliations:** 1 Department of Mechanical Engineering, Faculty of Engineering, Imperial College London, London, SW7 2AZ UK; 2 MSk Laboratory, Department of Surgery and Cancer, Faculty of Medicine, Imperial College London, London, W6 8PR UK; 3 Warwick Medical School, University of Warwick, Coventry, CV4 7AL UK; 4 Department of Medical Physics and Biomedical Engineering, University College London, London, WCIE 6BT UK; 5 Centre for Medicine, University of Leicester Medical School, Leicester, LE1 7HA UK; 6 Department of Mechanical Engineering, Faculty of Engineering, University of Moratuwa, Moratuwa, 10400 Sri Lanka; 7 St. Mary’s Hospital, North West London Major Trauma Centre, Imperial College, London, W2 1NY UK; 8 Diamond Light Source, Diamond House, Harwell Science and Innovation Campus, Didcot, OX11 0DE UK

**Keywords:** Structural biology, Biochemistry, Diseases, Trauma

## Abstract

Nanoscale mineralized collagen fibrils may be important determinants of whole-bone mechanical properties and contribute to the risk of age-related fractures. In a cross-sectional study nano- and tissue-level mechanics were compared across trabecular sections from the proximal femora of three groups (*n* = 10 each): ageing non-fractured donors (Controls); untreated fracture patients (Fx-Untreated); bisphosphonate-treated fracture patients (Fx-BisTreated). Collagen fibril, mineral and tissue mechanics were measured using synchrotron X-Ray diffraction of bone sections under load. Mechanical data were compared across groups, and tissue-level data were regressed against nano. Compared to controls fracture patients exhibited significantly lower critical tissue strain, max strain and normalized strength, with lower peak fibril and mineral strain. Bisphosphonate-treated exhibited the lowest properties. In all three groups, peak mineral strain coincided with maximum tissue strength (i.e. ultimate stress), whilst peak fibril strain occurred afterwards (i.e. higher tissue strain). Tissue strain and strength were positively and strongly correlated with peak fibril and mineral strains. Age-related fractures were associated with lower peak fibril and mineral strain irrespective of treatment. Indicating earlier mineral disengagement and the subsequent onset of fibril sliding is one of the key mechanisms leading to fracture. Treatments for fragility should target collagen-mineral interactions to restore nano-scale strain to that of healthy bone.

## Introduction

Bone fragility has long been attributed to a loss of mass, structure, mineral density and reduced tissue material properties^1,2^. However, few studies have ever investigated nano-level building blocks in fragile or fractured bone^3–7^, perhaps because of the technical difficulty of imaging bone nanomechanics^8^. At the nanoscale, the bone matrix is a composite of collagen fibrils and mineral platelets. An understanding of the role of collagen and mineral mechanics in fragility will be essential for developing effective diagnostics and interventions for preventing fractures^9^.

Bone matrix is a mesh of crosslinked collagen fibrils, coated and embedded with mineral platelets. Deformation mechanisms at the nanoscale may explain deformation and damage mechanisms at higher scales of bone’s hierarchical structure, including macroscale (i.e. whole-bone) deformation^10^ and fracture. Two key nanoscale bone deformation mechanisms in collagen and mineral have been suggested^10,11^ and both could contribute to bone strength or fragility.

The first involves the collagen fibrils (~ 100's nm long and 100 nm diameter^12^) sliding relative to each other when the interfaces between the individual fibrils are sheared beyond breaking point. The basis for this suggested mechanism is an experimental observation that when bone tissue is stretched the strain in nanoscale bone fibrils initially increases linearly with macroscale bone stretch but subsequently reaches a maximum or a plateau, and further stretching of the bone does not lead to a corresponding increase in fibril strain. Such a behaviour of the fibril strain under load may be explained as the fibrils sliding relative to each other^10^.

The second mechanism involves the mineral platelets (~ 50 nm long, 25 nm wide and 3 nm thick^12^) which behave similarly. Platelets start to slide relative to each other when the interfaces between the collagen and mineral are sheared beyond breaking point; in this paper, this process is called mineral disengagement. The basis for this suggested mechanism is an experimental observation that when bone tissue is stretched the strain in the nanoscale mineral initially increases but subsequently reaches a maximum and further stretching of the bone does not lead to a corresponding increase in mineral strain^10,11^.

The onset of fibril sliding and/or mineral disengagement under load could be a sign of the initiation of damage in bone, which eventually leads to fracture. The most comprehensive study investigating nanoscale deformation in human bone was carried out by Zimmerman et al., who reported that osteoporotic cortical tissue was prone to fibril sliding, whereas bone treated with bisphosphonate was less prone^13^. To date no studies have examined the mineral part of the matrix. It will be important to study the deformation of both components simultaneously to establish if the initiation of mineral disengagement occurred before or after the initiation of fibril sliding and which mechanism (if any) occurred simultaneously with maximum strength at the tissue level i.e., whether sliding or disengagement initiated the process of fracture.

Such comparisons between collagen and mineral deformation could be important for understanding the role of nanoscale bone mechanics in age-related fractures. In order to fully understand the role of nanoscale mechanics in age-related fractures, the next important research step will be to compare bone nano-mechanics between bone from donors with and without age-related fractures. Such a comparison would allow an assessment of the association between impaired nanoscale mechanics and age-related fractures.

## Aims

The main purpose of this study was to investigate the role of fibril sliding and mineral disengagement (represented by peak fibril and mineral strain respectively) in age-related fractures. In a cross-sectional study nano- and tissue-level mechanical properties were compared across trabecular bone samples from the proximal femora of three groups: ageing donors that had not suffered a fracture (Controls) and ageing fracture patients, both untreated (Fx-Untreated) and bisphosphonate-treated (Fx-BisTreated).

There were three objectives: (1) relate differences in the tissue-level mechanics of the three donor groups to differences in fibril sliding and mineral disengagement (represented by peak fibril and mineral strain respectively); (2) determine which mechanism, fibril sliding or mineral disengagement, initiated the process of fracture by comparing the onset of peak fibrillar, mineral and critical tissue strains (at peak tissue strength) across the three donor groups; and (3) test whether fibril sliding and mineral disengagement were correlated with tissue-level mechanics.

## Results

Supplementary Table 1 supplies the raw data for each bone sample including demographic, micro-CT, and mechanical information.

### Demographic and bone volume data

The median age of individuals in the Control, Fx-Untreated and Fx-BisTreated groups respectively was 73.0 (IQR 72.3–79.8), 80.0 (76.5–82.0) and 79.5 (76.0–82.0) (Table 1). There were no significant differences in age between the groups (Kruskal Wallis *p* = 0.135). The median bone volume fraction of the Control, Fx-Untreated and Fx-BisTreated groups respectively was 0.34 (IQR 0.33–0.36), 0.25 (0.24–0.26) and 0.26 (0.25–0.30) (Table 1). Controls possessed a significantly higher BV/TV than the Fx-Untreated and Fx-BisTreated groups and the fracture groups were not significantly different from each other (Kruskal Wallis *p* = 0.002 and pairwise Mann–Whitney *U* test Control vs. Fx-Untreated *p* = 0.001, Control vs. Fx-BisTreated *p* = 0.034, Fx-Untreated vs. Fx-BisTreated *p* = 0.251).

**Table 1:** Demographic and structural data for 3 groups: ageing donors that had not suffered a fracture (Controls) and ageing fracture patients, both untreated (Fx-Untreated) and bisphosphonate-treated (Fx-BisTreated).

Donor group	Donor number	Sex	Age (years)	BV/TV	Treatment (years)
Control	1	M	84	0.30	
	2	M	73	0.34	
	3	F	73	0.38	
	4	F	73	0.27	
	5	F	82	0.34	
	6	F	82	0.33	
	7	F	72	0.36	
	8	F	72	0.36	
	9	M	57	0.33	
	10	M	73	0.33	
Fx-Untreated	1	F	82	0.24	
	2	F	76	0.28	
	3	F	82	0.24	
	4	F	81	0.25	
	5	F	75	0.25	
	6	F	74	0.22	
	7	M	94	0.24	
	8	M	79	0.26	
	9	F	90	0.31	
	10	M	78	0.26	
Fx-BisTreated	1	F	88	0.29	9
	2	F	61	0.24	6
	3	F	79	0.26	5
	4	F	82	0.26	5
	5	F	84	0.37	5
	6	F	75	0.25	3
	7	F	82	0.42	2
	8	M	80	0.25	1
	9	F	68	0.23	1
	10	F	79	0.30	1

### Tissue versus nanomechanics

**Figure 1:**
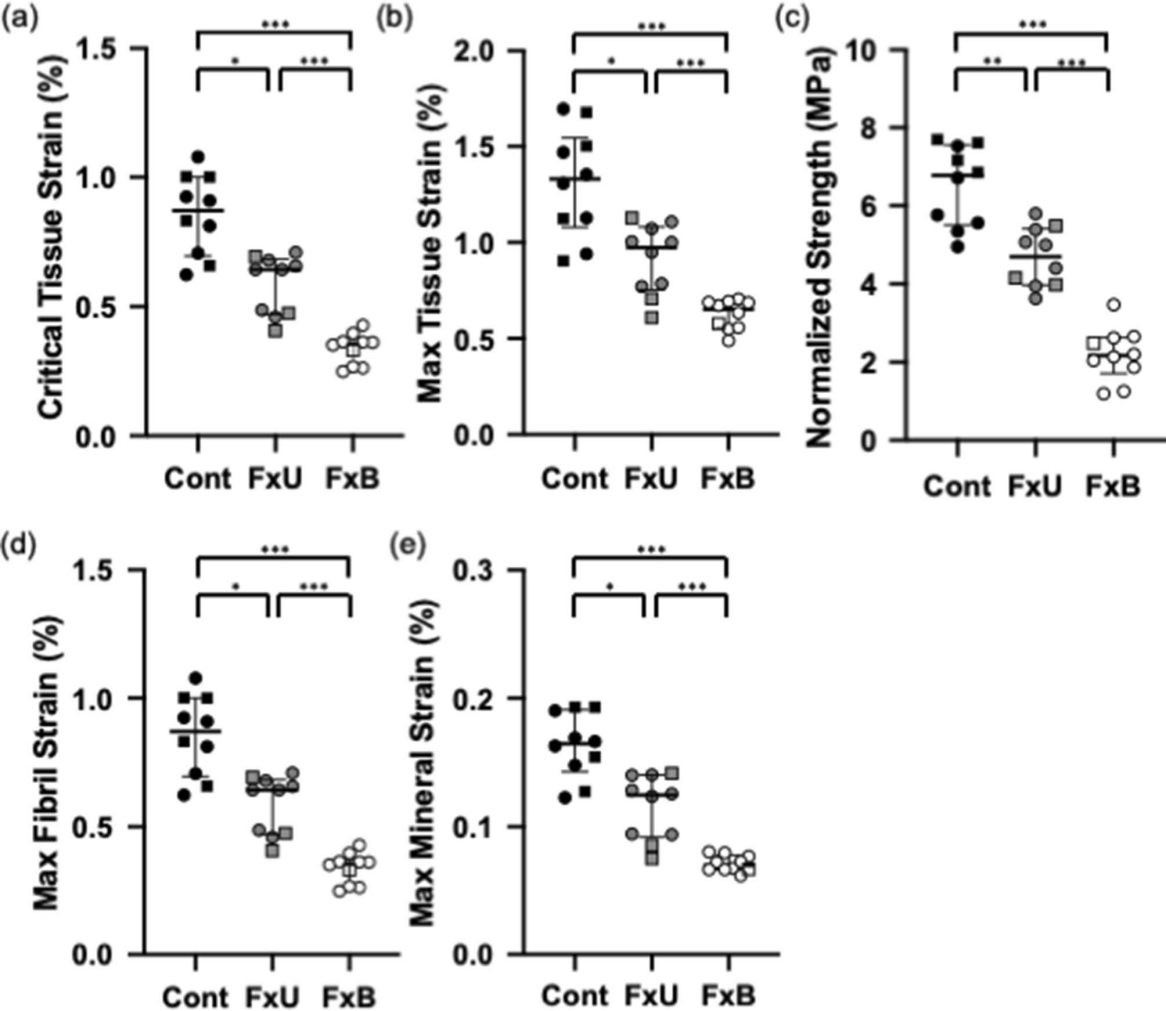
Comparison of bone tissue and nanomechanics across Control, Fx-Untreated and Fx-BisTreated groups (**a**) Critical tissue strain (**b**) Max tissue strain (**c**) Normalized strength (**d**) Max fibril strain (**e**) Max mineral strain. Kruskal Wallis (all *p* < 0.001) with pairwise Mann–Whitney *U* tests *p < 0.050, ** < 0.010 and *** < 0.001. Controls (filled black symbols), Fx-Untreated (filled grey symbols), and Fx-BisTreated (open symbols). Females shown as circles and males as squares.

The Control group exhibited the highest critical tissue strain (${\varepsilon }_{Tissue}^{nUTS})$ across all groups, followed by the Fx-Untreated and Fx-BisTreated groups, all of which were statistically significant Kruskal Wallis *p* < 0.001 (Fig. 1a). The Fx-BisTreated group exhibited the lowest critical tissue strain (${\varepsilon }_{Tissue}^{nUTS})$.

The Control group exhibited the highest maximum tissue strain across all groups, followed by the Fx-Untreated and Fx-BisTreated groups, all of which were statistically significant Kruskal Wallis *p* < 0.001 (Fig. 1b). The Fx-BisTreated group exhibited the lowest maximum tissue strain.

The Control group exhibited the highest strength across all groups, followed by the Fx-Untreated and Fx-BisTreated groups, all of which were statistically significant Kruskal Wallis *p* < 0.001 (Fig. 1c). The Fx-BisTreated group exhibited the lowest tensile strength. Figure 1c presents the strength normalized for BV/TV as described in the method. The variation of raw strength with BV/TV is provided in Supplementary Fig. 1.

The Control group exhibited significantly higher peak fibril strain (Fig. 1d) and peak mineral strain (Fig. 1e) followed by the Fx-Untreated and Fx-BisTreated groups Kruskal Wallis *p* < 0.001. The Fx-BisTreated group exhibited the lowest peak fibril and mineral strains.

### Critical tissue strain versus peak fibril and mineral strains

**Figure 2:**
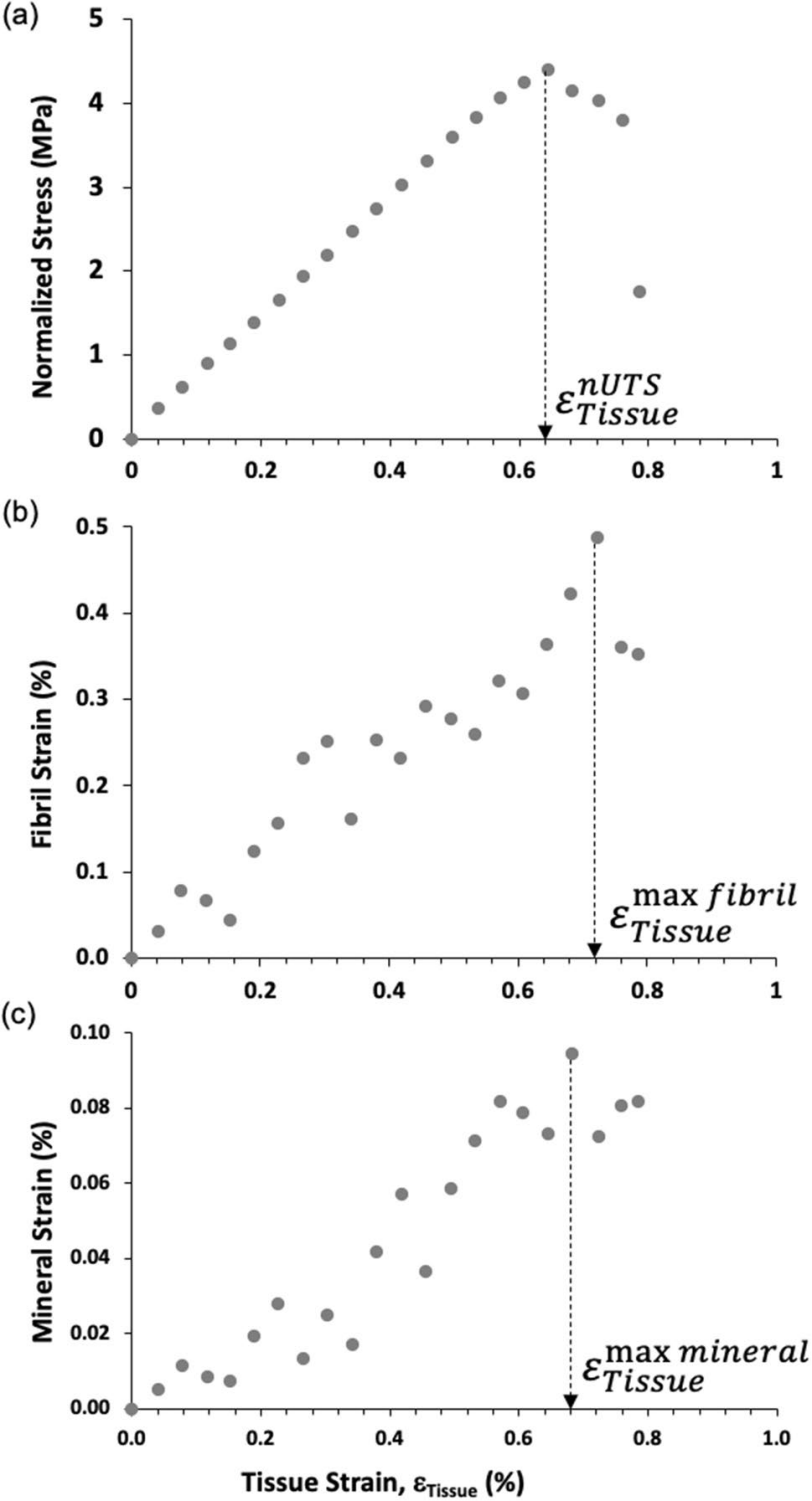
Local displacement curves for a single typical bone sample. (**a**) Macroscale stress–strain curve indicating the macroscale property of critical tissue strain ($\varepsilon_{Tissue}^{nUTS}$). (**b**) Nanoscale fibril-tissue strain curve showing tissue strains to reach the nanoscale property of peak fibril strain ($\varepsilon_{Tissue}^{\max \;fibril}$). (**c**) Nanoscale mineral-tissue strain curve showing the tissue strains to reach the nanoscale property of peak mineral strain ($\varepsilon_{Tissue}^{\max \;mineral}$). Tissue strain is plotted along the x-axis in all three figures allowing a comparison of the occurrence of key events (the peaks) from the nanoscale to the macroscale.

**Figure 3:**
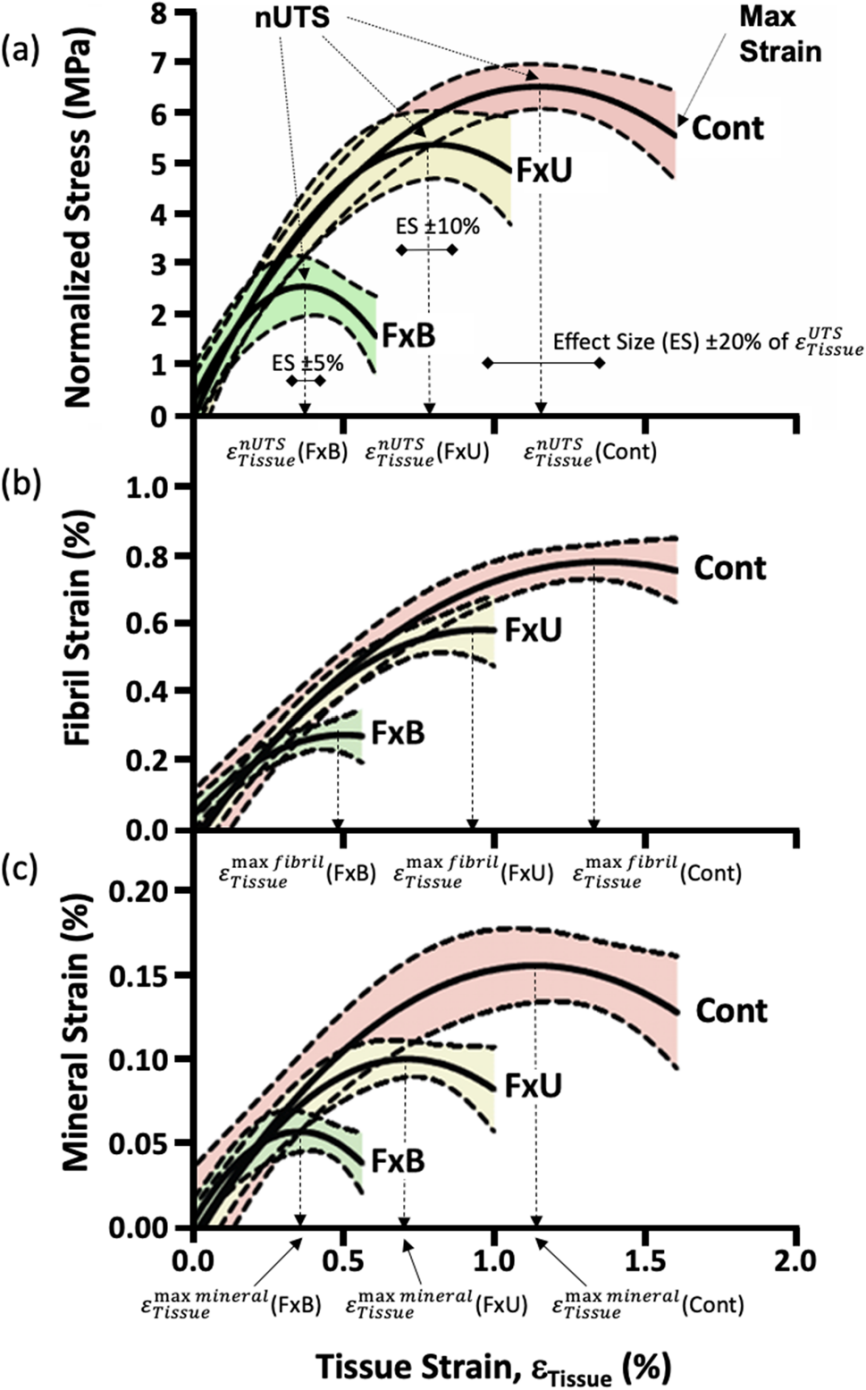
Overall stress and strain curves for Control, Fx-Untreated and Fx-BisTreated groups. (**a**) Normalized stress–strain curves indicating the critical ($\varepsilon_{Tissue}^{nUTS}$) and max tissue strains for each group. Effect sizes (ES) used in the statistical analyses are shown (see Table 2). (**b**) Fibril-tissue strain curves showing tissue strains to reach peak fibril strain ($\varepsilon_{Tissue}^{\max \;fibril}$). (**c**) Mineral-tissue strain curves showing the tissue strains to reach peak mineral strain ($\varepsilon_{Tissue}^{\max \;mineral}$). Shaded areas represent the 95% confidence intervals for *n* = 10 donors per group.

Stresses and strains were plotted for each of the 30 samples i.e., 10 in each of the 3 groups (Fig. 2). The amalgamation of all 30 data sets is shown in Fig. 3. A least mean square regression on a quadratic model of the data and including 95% confidence intervals was applied to each of the 3 groups and the results of this analysis are shown in Fig. 3. Statistical analyses were calculated using GraphPad Prism 8 (San Diego, California).

The critical tissue strain, peak fibrillar strain and peak mineral strain were all compared across the three donor groups. The critical tissue strain ${\varepsilon }_{Tissue}^{nUTS},$ peak fibril strain ${\varepsilon }_{Tissue}^{fibril}$ and peak mineral strain ${\varepsilon }_{Tissue}^{mineral}$ are highlighted in Fig. 3 by arrows with dashed lines.

The critical tissue strain (i.e. the point at which the bone sample reached maximum tensile strength and maximum load-carrying capacity) coincided with the peak mineral strain. Note that for all three donor groups the tissue strains at ${\varepsilon }_{Tissue}^{nUTS}$ in Fig. 3a and ${\varepsilon }_{Tissue}^{mineral}$ in Fig. 3c were similar.

In contrast, the peak strain in the collagen fibrils occurred after both the critical tissue strain and the peak mineral strain, but before the maximum tissue strain (i.e. the point at which the bone sample fractured). Note that for all three donor groups the tissue strain at ${\varepsilon }_{Tissue}^{fibril}$ in Fig. 3b was higher than the tissue strain at ${\varepsilon }_{Tissue}^{nUTS}$ in Fig. 3a and ${\varepsilon }_{Tissue}^{mineral}$ Fig. 3c. Further, the tissue strain at ${\varepsilon }_{Tissue}^{fibril}$ in Fig. 3b was lower than the max tissue strain in Fig. 3a. Hence peak fibril strain occurred in the region of plastic deformation (i.e. after the bone sample reached the critical strain at nUTS but before reaching the maximum tissue strain).

The tissue strain values at $${\varepsilon }_{Tissue}^{nUTS},$$$${\varepsilon }_{Tissue}^{fibril}$$ and $${\varepsilon }_{Tissue}^{mineral}$$ were compared directly in Table 2. In all 3 groups, the critical tissue strain ($${\varepsilon }_{Tissue}^{nUTS}$$) and the tissue strain at peak mineral strain ($${\varepsilon }_{Tissue}^{\max \; mineral}$$) were identical i.e., within 1%. Whilst the tissue strain at peak fibril strain was higher (5–14%). The differences in strain between $${\varepsilon }_{Tissue}^{nUTS}$$, $${\varepsilon }_{Tissue}^{\max \; fibril}$$ and $${\varepsilon }_{Tissue}^{\max \; mineral}$$ were not significant but approached significance for $${\varepsilon }_{Tissue}^{nUTS}$$ versus $${\varepsilon }_{Tissue}^{\max \; fibril}$$ (see Table 2).

**Table 2:** Association between the critical tissue strain ($\varepsilon_{Tissue}^{nUTS}$), max fibril ($\varepsilon_{Tissue}^{\max \;fibril}$) and max mineral ($\varepsilon_{Tissue}^{\max \;mineral}$) strain respectively.

	$\varepsilon_{Tissue}^{nUTS}$(%)	$\varepsilon_{Tissue}^{\max \;fibril}$(%)	$\varepsilon_{Tissue}^{\max \;mineral}$(%)	ANOVA *p*	$\varepsilon_{Tissue}^{nUTS}$ vs. $\varepsilon_{Tissue}^{\max \;fibril}$ *p*	$\varepsilon_{Tissue}^{nUTS}$ vs. $\varepsilon_{Tissue}^{\max \;mineral}$ *p*	$\varepsilon_{Tissue}^{\max \;fibril}$ vs. $\varepsilon_{Tissue}^{\max \;mineral}$ *p*
Control	1.04 (0.30)	1.18 (0.30)	1.05 (0.28)	0.056	0.076	0.985	0.105
Fx-Untreated	0.72 (0.19)	0.78 (0.22)	0.73 (0.19)	0.084	0.097	0.948	0.168
Fx-BisTreated	0.48 (0.10)	0.55 (0.05)	0.48 (0.10)	0.050	0.075	0.994	0.088

In all 3 groups the critical tissue strain is more closely approximated by the tissue strain at max mineral strain (1%) than the tissue strain at max fibril strain (5–14%). Mean and (StDev) compared using paired ANOVA with Tukey’s post hoc.

Note that the graphs of Fig. 3 were intended to summarise the data for the reader and were created by combining the data points from all 30 samples, whereas Fig. 1 and Table 2 were created by assessing the mechanical response of the 30 samples from individual graphs of each specimen, such as shown in Fig. 2. Hence, the values in the box plot Fig. 1 are slightly different from those in the curves depicted in Fig. 3. However, the principal findings are consistent between Figs. 1 and 3. At the tissue level as well as the nanoscale, the Fx-BisTreated tissue had lower mechanical properties than Fx-Untreated donors which in turn were lower than the Control.

### Regression analysis of tissue and nano and level mechanics

**Figure 4:**
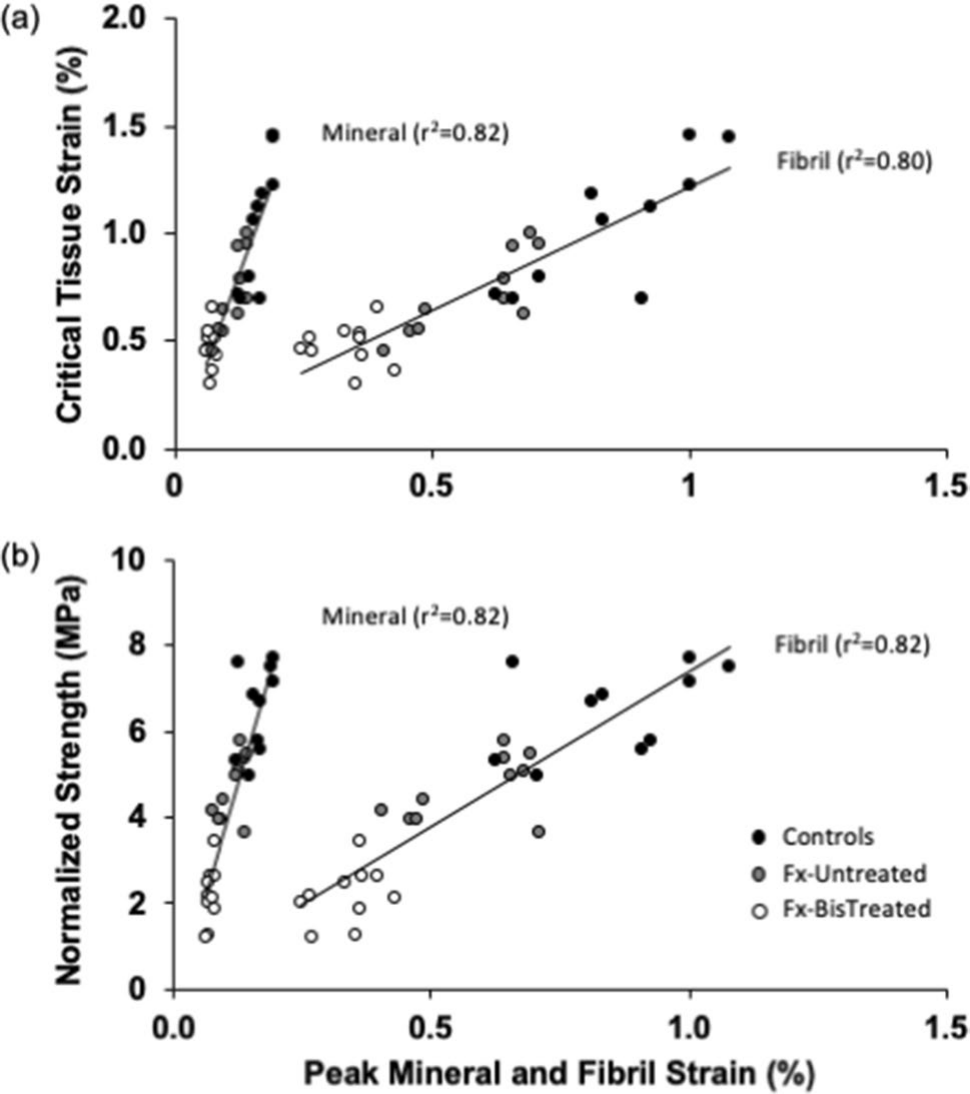
Tissue-level mechanical properties are positively and strongly correlated with peak fibril and mineral strain for both (**a**) critical tissue strain and (**b**) normalized strength.

The critical tissue strain (${\varepsilon }_{Tissue}^{nUTS}$) was positively correlated with both the peak fibril (*r*^2^ = 0.80) and mineral strain (*r*^2^ = 0.82) (Fig. 4a). Similarly, the strength values were positively correlated with both the peak fibril (*r*^2^ = 0.82) and peak mineral strain (*r*^2^ = 0.82) (Fig. 4b).

## Discussion

Nano- and tissue-level mechanical properties in the proximal femora were compared between patients that had a fractured neck of femur (with either a bisphosphonate treatment history or without) and fracture-naive individuals (collected from a tissue donor bank). As expected the tissue level properties including critical strain (Fig. 1a), maximum strain to failure (Fig. 1b) and strength (Fig. 1c) were significantly lower in the untreated and bisphosphonate-treated fracture groups (Fx-Untreated and Fx-BisTreated) than in the ageing non-fracture group (Control). The comparison of nanoscale properties was consistent with macroscale properties. Peak strain measured in the fibrils and mineral was significantly lower in the fracture groups than the Control (Fig. 1d,e). Overall the Fx-BisTreated group exhibited the lowest critical tissue strain, maximum strain to failure and strength of the fracture groups (Fig. 1a–c), as well as lowest peak fibril (Fig. 1d) and mineral strain (Fig. 1e), despite the Fx-BisTreated and Fx-Untreated sharing similar BV/TV. Across all three groups, critical tissue strain and strength were strongly and positively correlated with the nanoscale strains (Fig. 4). The critical tissue strain and strength increase with increasing deformability at the nano-level. Overall the data support the conclusion that peak fibril and mineral strain are important determinants of tissue-level mechanical properties and contribute to reduced critical strain and bone strength in patients with age-related hip-fractures.

Results must be interpreted with caution because these are observational, cross-sectional data with all the potential selection biases that cannot be adjusted for. The pattern of decreasing critical strain and strength at the tissue level and strain at the nano level from Control to Fx-Untreated and Fx-BisTreated groups (Fig. 1a) suggest that fractures are associated with altered material properties at the nanoscale, which contribute to loss of whole-bone strength and therefore increased bone fragility. This interpretation is supported by the positive correlation between tissue-level properties (critical strain and strength) with both peak fibril and peak mineral strain (Fig. 4). Taken together the data suggest there could be a strong relationship between nano-, tissue- and therefore whole-bone mechanical properties in the femur.

### Bisphosphonates might reduce mineral strain

Our finding that bone from the Fx-BisTreated group exhibited the lowest critical strain and strength compared to the bone from the Fx-Untreated and Control (Fig. 1a) contributes to the current debate about the effect of bisphosphonate treatment on bone mechanical properties. One possible explanation is that bisphosphonates might reduce mechanical properties by binding to the mineral phase and reducing the mineral’s ability to deform. Evidence for this suggestion comes from experiments where mouse bones soaked in zoledronate for 48 h were found to exhibit reduced work to failure under mechanical testing^14^ in comparison to controls. Half of the Fx-BisTreated donors published in this study had been prescribed oral alendronate for between 5 and 13 years (Table 1), which would be considered long-term therapy. Assuming the patients adhered to the prescriptions and took the oral pill correctly. Most clinical guidelines recommend a treatment review should be performed after 5 years of continuous therapy^15,16^. Therefore, one plausible explanation for the relatively poor performance of the Fx-Bistreated group is that although treatment yields improvements in BMD and porosity, following long-term therapy these factors may be offset by nanostructural changes resulting in reduced mineral and fibril capacity to sustain high strain.

It should be noted that there are alternative interpretations of the bisphosphonate data. The reduced nano- and macro-mechanics in the Fx-BisTreated group compared to the Fx-Untreated could reflect other aspects of bone health that affect fragility, for example, mineral density, bone mass or microarchitecture. Fracture groups had similar BV/TV (Table 1) so microarchitecture is unlikely to account for the low strength of Fx-BisTreated patients. Bisphosphonate treatment suppresses bone remodelling and has been associated with an accumulation of micro-cracks which reduce bone strength^17^. However, cross-sectional, or preferably longitudinal studies of the association between bisphosphonate treatment duration and nanomechanics must fully elucidate the effects of therapy at the nanoscale.

### Mineral component governs bone strength and failure

Perhaps the most interesting finding of the study is that the maximum strain in the apatite mineral ${(\varepsilon }_{Tissue}^{\max \; mineral})$ coincided with the peak stress at the macroscale (${\varepsilon }_{Tissue}^{nUTS}$) (Fig. 3a,c, Table 2). This observation implies that when the mineral no longer efficiently contributed to the load-carrying capacity of the matrix (i.e. mineral disengaged), the bone sample also lost load-carrying capacity and failed. Thus, mineral disengagement may play a central role in the macroscopic strength of bone tissue.

### Fibril component resists fracture

In contrast fibril strain peaked (${\varepsilon }_{Tissue}^{\max \; fibril})$ in the range of plastic deformation i.e., just after the peak stress at the macroscale (${\varepsilon }_{Tissue}^{nUTS}$) and well before the bone section fractured (maximum tissue strain) (Fig. 3a,b, Table 2). Thus, fibril sliding, and the damage associated with fibril sliding, seems to represent an early stage in the cascade of events that eventually led to the fracture of the bone on the macroscale.

Energy is required to plastically deform bone beyond the peak strength (i.e. nUTS) until a bone sample fractures (i.e. area under the curve Fig. 3a). This energy is spent making the collagen fibrils slide relative to each other which in turn is the source of the plastic deformation. As a result, the bone tissue is better able to absorb the impact energy during a bump or fall, potentially preventing whole-bone fracture. Hence it seems that while mineral disengagement dominates the load-carrying capacity (i.e. strength) the fibril sliding could be an important mechanism for absorbing energy (during plastic deformation) to stop the bone from fracturing.

### Age-related fractures associated with early mineral disengagement and fibril sliding

Both mineral disengagement and fibril sliding happened earlier (i.e. at lower tissue strain levels) in the Fx-BisTreated and Fx-Untreated groups relative to the Control group (Fig. 3). Therefore, irrespective of treatment fractured bone was more prone to mineral disengagement, fibril sliding and early damage. Which explains the lower critical tissue strain, maximum strain and strength of fractured bone on the macroscale when compared to controls (Figs. 1 and 3).

### Role of mineral disengagement and fibril sliding in bone fracture

The association between peak mineral and fibril strains with bone failure and bone fracture respectively were consistent across the three donor groups (Control, Fx-Untreated and Fx-BisTreated). This suggests that the mechanisms which led to reduced strength and fracture involve a reduction in the ability of the mineral phase to contribute to the load-carrying capacity of the bone causing mineral disengagement, then an early onset of fibril sliding, and eventually fracture. Our novel data supports disengagement of apatite mineral from the collagen fibrils is the initiating event of bone failure. At the strains observed in this study, this disengagement could involve separation of the mineral from the fibril and perhaps fracturing of the mineral. Since mineral disengagement and subsequent fibril sliding happen more readily in fractured patients, regardless of treatment status, this could be a clinically important mechanism.

### Nano strain influence on whole-bone strength

The central finding of the study is that irrespective of treatment, age-related fractures are associated with reduced deformability of bone-matrix at the nanoscale (Fig. 1). Further, critical strain and strength at the tissue scale are strongly positively correlated with the fibrillar and mineral strain (Fig. 4). Hence it is likely that nano-strains do play a key role in determining whole-bone strength or fragility. Nano-mechanical properties might reduce macroscopic strength, either directly by limiting the ability of a bone to bend and absorb energy during a trip or fall, and/or indirectly by reducing the energy required to form micro-cracks in the collagen-mineral matrix^17,18^.

### Nanoscale fracture initiation

Previous studies have reported a higher micro-crack density in the bone of fracture patients relative to controls^17,18^. Reduced nano-mechanical performance in bone from fracture patients may be the mechanism. Mineral disengagement and fibril sliding may result in nanoscale fractures, which then propagate to form micro-cracks at the tissue scale, and, eventually, whole-bone fracture. The reduced nano-mechanical performance of Fx-Untreated and Fx-BisTreated groups may have led to crack initiation at lower macro stresses or strains than Controls, thereby increasing the risk of a fracture nucleating under traumatic load.

### Nanostructure and mechanics

The nanoscale brittleness of fracture patients (Fx-Untreated and Fx-BisTreated) found in this study could be due to an increase in the number of AGE crosslinks between collagen fibrils and/or mineral crystal thickness as has been previously reported with age^5,19,20^ and bisphosphonate therapy^21,22^. Such correlations between compositional characteristics and mechanical properties do not prove causation but simple mechanisms could be at work. Crosslinks might reduce the ability of collagen fibrils to slide^10,23,24^, thereby reducing the strain for a given load^24^. Similarly, thicker crystals may deform less under load and so exhibit reduced strain^25,26^ and limit bone strength^4,27,28^.

Increased cross-linking and mineral crystal size could also reduce fracture toughness^29^ and affect the ability of bone to form stable microdamage or predispose the bone to premature microcracks that in subsequent load cycles may initiate whole-bone fracture. Stable damage formation is related to higher toughness and resistance to fracture in bone^30^ while high levels of pre-existing microcracks in Fx-BisTreated treated bone has been related to low strength^17,18^. Hence, the low strength of Fx-Untreated and Fx-BisTreated treated bone found in this study may be due to a reduced ability to sustain stable damage formation and/or high levels of pre-existing microcracks which in turn might have contributed to the neck of femur fracture. There is a possibility that long-term bisphosphonate could further exacerbate microdamage accumulation by suppressing remodelling and therefore removal of cracks^17,31,32^.

### Nanomechanics: a new component of age-related bone fragility

Our results on the mechanical properties of bone indicate that (in addition to a loss in bone mass) age-related fractures are associated with poor material i.e., inferior nano-mechanical properties which in turn lead to lower toughness and microcracking (i.e. inability to sustain stable microdamage). Bone remodelling is targeted to microcracks^33^ and osteoclastic resorption cavities could cause a stress concentration in the tissue leading to more microcracks, more resorption and eventually perforation of trabeculae^34^. Focal defects lead to loss of connectivity within the overall bone structure and compromise bone strength more significantly than universal thinning. Therefore, impaired nanoscale mechanics could accelerate and focus bone loss leading to a large loss of trabecular strength for minimal loss of mass. To fully understand the mechanisms behind bone fragility fractures and this nano- and micro-mechanical aspects of bone quality need to be investigated in addition to effects on bone mass, structure and metabolism. These studies should include detailed analysis of cross-links, mineral dimensions, collagen-mineral interface, mineral disengagement, fibril sliding, diffuse damage, microcracks, remodeling, perforations, bone mass and fractures.

### Limitations of synchrotron scattering and diffraction

Papers publishing bone diffraction experiments often overlook a potentially major limitation of the method which is the small size of the region investigated by the X-ray beam (i.e. beam incident on the sample was 200 μm diameter). Other mechanisms (such as damage occurring outside the beam) could off-load the investigated region suggesting an alternative explanation for the plateauing of fibre and mineral strains seen in Fig. 3. However, if this was the case, the tissue strain to fibril sliding,$({\varepsilon }_{Tissue}^{\max \; fibril}$), and tissue strain to mineral disengagement ${(\varepsilon }_{Tissue}^{\max \; mineral})$, would have been the same for each group of donors. However, for each of the three donor groups, the ${\varepsilon }_{Tissue}^{\max \; fibril}$ was higher than ${\varepsilon }_{Tissue}^{\max \; mineral}$, although not significantly so. Thus, an offloading mechanism is unlikely to account for our data.

Further, the reliability of our nanomechanical measurements is supported by a comparison of our data with other work. We found a mean maximum fibril strain in the Fx-Untreated group of 0.59% (Fig. 1d). In similar studies using SAXD measurements of fibril strain, Gupta and colleagues^10^ reported a maximum strain of around 0.40% while Zimmermann and colleagues^13^ reported a maximum strain of approximately 0.70%. Gupta et al*.* used bovine bone while Zimmermann et al.,^13^ used osteoporotic human cortical bone. Bovine bone is stiffer than human bone which may explain the relatively low strains reported by Gupta and colleagues^10^. Using WAXD, Gupta and colleagues^10^ also reported maximum mineral strain in the range of 0.15 to 0.20% which is consistent with the mean maximum mineral strain of 0.16% found in this study for the Control group (Fig. 1e). Given that all these studies imaged small regions (a few hundred microns) of a sample and the strain data was similar the measures are likely to be representative of the whole bone.

Our findings were not in complete agreement with the literature concerning the effects of disease and bisphosphonate therapy. Zimmermann and colleagues^13^ found a reduction in maximum fibril strain (i.e. a drop in the onset of fibril sliding) of approximately 40% due to osteoporosis. Similarly, we found a 31% reduction in maximum fibril strain of the Fx-Untreated group compared to the Controls group. However, in complete contrast, we found a drop of 60% in the bisphosphonate-treated group while Zimmermann and colleagues^13^ reported that young and bisphosphonate-treated bone had similar onset of fibril sliding and essentially that bisphosphonates had restored the nanomechanical properties of the bone.

The dramatically different findings could be due to the type of tissue because Zimmerman and colleagues tested cortical bone and the mechanical data presented here are from the trabecular bone. Local characteristics of the microarchitecture including porosity, trabecular dimensions, and organisation, could cause the failure process to be more stochastic, especially in the small samples used by both studies. Further, tissue structure might also affect the distribution and penetration of bisphosphonates^35^. Trabecular bone has a higher surface area to volume ratio and is remodelled at higher rates so bisphosphonates might be more concentrated than in cortical tissue. Hence the effect of bisphosphonates on mechanics of trabecular and cortical tissue might also vary.

A final issue that must be considered is the accuracy at which the timing of peak mineral and fibril strains can be measured in the mineral and fibril strain curves (see Fig. 2). The steps in tissue strain are 0.03% (± 0.003 SD), which is the limit for accuracy. The differences between peak mineral and fibril strains were larger i.e., 0.13, 0.05, and 0.07 for the three groups (see Table 2). Hence the strain data did resolve the timing of mineral and fibril peaks.

### Nanoscale insights into the material basis of fragility

In the clinical and public consciousness, skeletal fragility is due to osteoporosis i.e., loss of bone mineral density (BMD). Therefore, measurement of BMD is currently the most commonly used densitometric technique for quantifying bone fracture risk and starting bisphosphonate treatment. Usually, the areal bone mineral density (i.e. aBMD) is assessed using a DEXA scan which probably measures the bone mass along the direction of the X-ray projection^36^. Though widely used in clinical practice a lot of evidence suggests that it is not possible to confidently predict who will fracture or track the benefits of bisphosphonate therapy using DEXA.

Although there is some correlation between decreasing aBMD and an increased fracture risk^37^, more than 50% of fragility fractures occur in patients with a normal aBMD^38^. Indeed, low bone mineral density (T-score <  − 2.5) is not sufficient to predict almost half of the non-vertebral fractures (including hip) in postmenopausal women^39^. Notwithstanding the finding that there is a false positive rate of about 15% which probably leads to unnecessary anxiety and therapeutic prescriptions^40^.

More than a decade ago a more nuanced understanding that skeletal fragility is complex and crosses many hierarchical levels, from macro- to micro- and nano-scale was described^41^. It was thought that nanoscale effects on whole-bone mechanics are independent of the micro and macro-scale. The data presented here support the theory that age-related fractures are directly dependent on mechanical properties at the nanoscale. We propose ageing at bone nanoscale causes changes in the microdamage, microarchitecture and bone geometry that contribute to hip-fractures: via the mechanisms set out above.

Therefore, bone health assessments based on bone material and nanoscale mechanics might have greater sensitivity and specificity than current tests for diagnosing bone fragility, and higher accuracy for predicting fracture risk which would improve treatment decisions. Hence it is important future research continues to investigate the nanoscale material basis for bone strength and most importantly the knock-on effects of ageing at the nanoscale on the micro- and macro-scale.

It has long been known that mineral plays a key role in bone strength and it is not just mass or distribution that is important, but rather the mechanical properties of the mineral platelets (and crystals) and the interactions with the collagen fibrils are key. The study of these interactions is a new frontier in bone research.

## Conclusion

Age-related fractures were associated with impaired bone nanomechanics i.e., weaker mineral apatite that failed and debonded from the collagen matrix at lower stress leading to earlier fibril sliding and plastic deformation. Mineral strain and fibril strain were directly related to reduced critical strain and strength at the tissue-level which were lower in fractured patients, regardless of treatment status. Overall the data support the conclusion that reduced peak fibril and mineral strain are important determinants of tissue-level mechanical properties and contributed to reduced bone deformability and strength in patients with age-related hip-fractures. Therefore, bone nanomechanics is potentially a clinically important component of age-related fragility fractures.

The study demonstrates the urgent need for research investigating the role of nanostructure in whole-bone strength and the importance of the interconnection between mineral and collagen and other factors that may affect the nanomechanics of bone. Studies investigating how nanomechanics is related to the ability of bone to sustain stable microdamage and to the introduction of pre-existing cracks in the bone are also warranted.

Our study adds a new dimension to the fundamental understanding of the pathophysiology of bone fragility and the effects of bisphosphonate treatment. Highlighting the need to move away from the conventional approaches based on bulk bone mineral or mass towards utilizing information about the structural hierarchy and how mechanical properties at each level contribute to strength or fragility. Potentially opening avenues for new diagnostic tests and therapies which will address the nanoscale contribution to fragility. Novel fracture risk treatments could target the advanced glycation endproducts (AGE) collagen crosslinks, the mineral dimensions, and possibly the connections between the mineral and collagen to restore or delay the onset of mineral disengagement and subsequent fibril sliding to match that of healthy bone.

## Materials and method

### Sample information

Ethical approval was granted by the Imperial College Tissue Bank (R13004) and all patients consented for permission to use excess tissue from hip arthroplasty in research. All procedures performed in this study were in accordance with the ethical standards of the institutional and national research committees and with the 1964 Helsinki Declaration and its later amendments. Patients’ histories of metabolic bone disease and treatment were collected from patient records. All individuals with a history of primary bone disease or underlying disorders such as cancer, which could lead to secondary bone disease were excluded from this study.

A consecutive series of femoral heads were collected from 21 patients with an intracapsular fracture after written informed consent was provided at St Mary's Hospital (London, United Kingdom) between May 2015 and September 2017. In total, 20 patients were included in the study and one was excluded due to avascular necrosis. Patients were divided into those who had been prescribed bisphosphonate therapy and those who had not. Femora from non-fracture donors came from cadavers donated to the Biological Resource Centre (AZ, USA). Medical records and physical assessments were examined to select 10 donors with no history of fractures, bone metabolic disease or treatments. It is important to note that it was not possible to obtain dual-energy X-ray absorptiometry (DXA), fracture risk assessment tool (FRAX) or body mass index (BMI) data for any of the donors.

In total, 30 donors were divided into three groups: an ageing non-fracture control group (Control), an untreated fracture group (Fx-Untreated), and a bisphosphonate-treated fracture group (Fx-BisTreated). Demographic data including age, sex and treatment duration are presented in Table 1.

### Sample preparation

**Figure 5:**
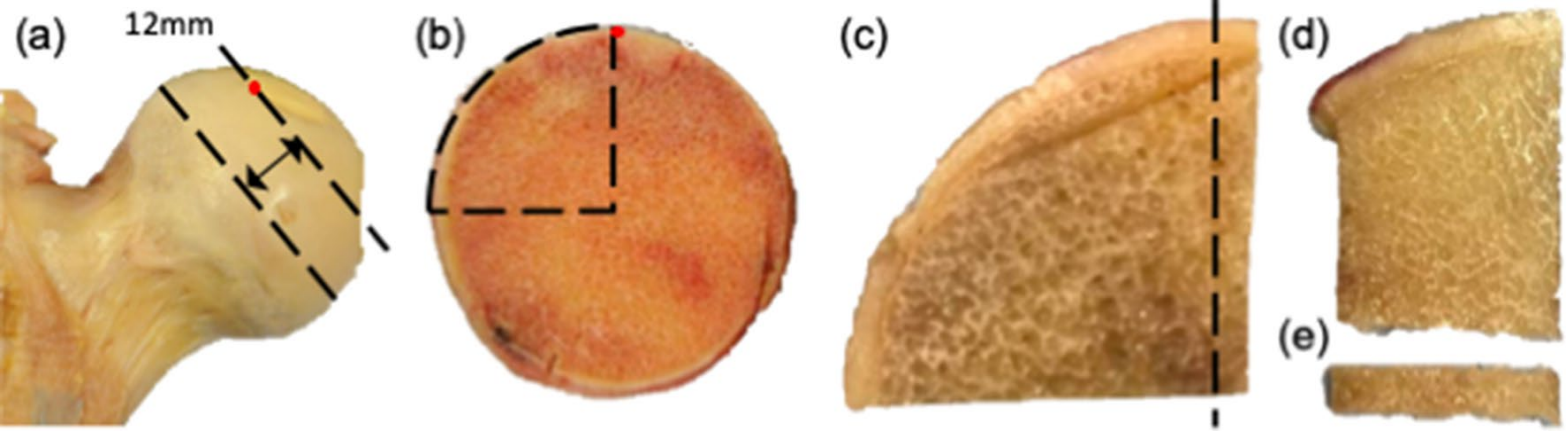
Preparation of bone sections for tensile testing along the axis of the femoral neck (black arrow). (**a**) Femoral slice cut from the widest point of the femoral head 12 mm thick. (**b**) Arc sectioned using two more cuts, one along the edge of the fovea capitis (used as a reference point) and one at 90°. (**d**) Edge piece sectioned from the arc facing fovea and (**e**) section cut from the end closest to the chiasma along the axis of the femoral neck (12.0 × 2.8 × 1.0 mm).

Thirty rectangular-shaped standard tensile testing samples were prepared, one from each donor. These samples were sectioned from the region directly superior to the trabecular chiasma in the primary tensile trabecular arcade of the femoral heads (Fig. 5). An initial cut was made at the widest point of the femoral head and a second cut 12 mm proximally. Subsequently, an arc-shaped piece was sectioned by making two more cuts; one along the edge of the fovea capitis (used as a reference point) and one at 90°. A slice was taken from the edge facing the fovea and finally the tensile section cut from the end closest to the chiasma. Each tensile section was 12.0 mm in length, 2.8 mm in width and 1.0 mm in thickness. The small thickness of the samples was necessary to enable accurate x-ray diffraction measurements. The ends of each specimen were potted into a 3D printed holder using dental cement, which served as clamps during the testing and resulted in a gauge length of 6 mm. The specimens were stored at -80ºC and only removed from storage for tensile testing.

### Micro-CT scanning

Before mechanical testing all 30 of the tensile trabecular sections were imaged using a micro-CT system (Nikon X-Tek HMXST-225: tungsten target; X-Ray beam 180 keV and 200 µA, voxel size 30 µm; 3,124 projections; 360° rotation). Total scan time was 30 min. Scans were used to calculate the bone volume fraction (BV/TV) for the entire tissue section (not just the region which would be imaged using scattering and diffraction). Scans contained 256 grey values and were segmented into binary images using the grey value frequency distribution plot to apply a global threshold at the minima between the two peaks that represented marrow and bone^42–44^. The advantage of this method is that variations in bone density or scan performance are taken into account and do not affect structural measurements (e.g. BV/TV). The minima at which the threshold was placed ranged between 190 and 233 for the Controls, 200–225 for the Fx-Untreated and 206–222 for Fx-BisTreated. All of the voxels above the threshold were defined as bone and the values below as background. Bone volume fraction was calculated from the binary images using the BoneJ plugin for ImageJ (v1.49) by dividing the total number of voxels that represented bone by the total number of voxels in the whole scan volume.

### Tensile testing with simultaneous measurements of fibril and mineral strain

**Figure 6:**
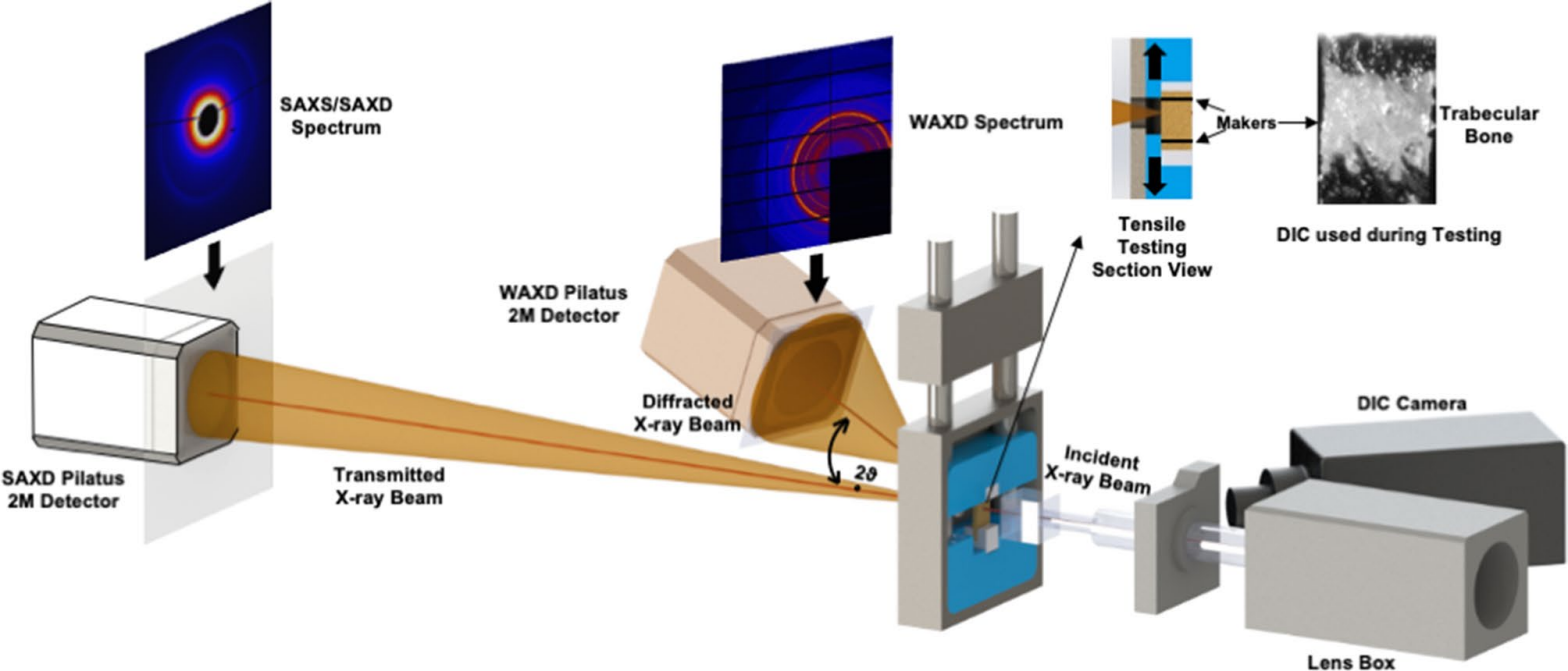
Schematic diagram of the synchrotron experiment showing both SAXD and WAXD setups to capture X-ray diffraction patterns. A high-speed camera tracked the movement of a tissue sample marked with two black lines for automatically calculating displacement.

A customized micromechanical testing rig, including a load cell, was used for the experiments. The testing rig was fully calibrated to the I22 Beamline facility settings at Diamond Light Source, United Kingdom, as used previously in other studies^45,46^ (Fig. 6). Tensile testing to failure was displacement-controlled, with a strain rate of 0.001 s^−1^. Specimens were defrosted for 30 min before testing then all tests were conducted at room temperature and the specimens were kept hydrated throughout. A high-speed camera was placed beside the testing rig to track the movement of two ink markers on the testing sample (Fig. 6). The tissue strain in each bone sample was calculated from the relative movement between these two markers. The apparent stress was calculated by dividing the measured load by specimen cross-sectional area and then normalized to remove variation attributable to bone volume by dividing by BV/TV to the power of 1.26^47^.

Tissue-level tensile mechanical properties of each bone sample were calculated by plotting the stress data against strain (Fig. 2a). The load–displacement plots were used to determine the critical tissue strain at maximum load-carrying capacity (${\varepsilon }_{Tissue}^{nUTS}$), the maximum tissue strain to fracture and the normalized ultimate tensile strength (nUTS). Critical tissue strain, rather than the normalized ultimate tensile strength, was the key property used to compare the donor groups (and relate the macromechanics to nanomechanics) as strain is relatively insensitive to differences in BV/TV between groups^19^. However, critical tissue strain and normalized ultimate tensile strength (henceforth referred to as strength) were both included to explain the findings of this study to a broad audience.

**Figure 7:**
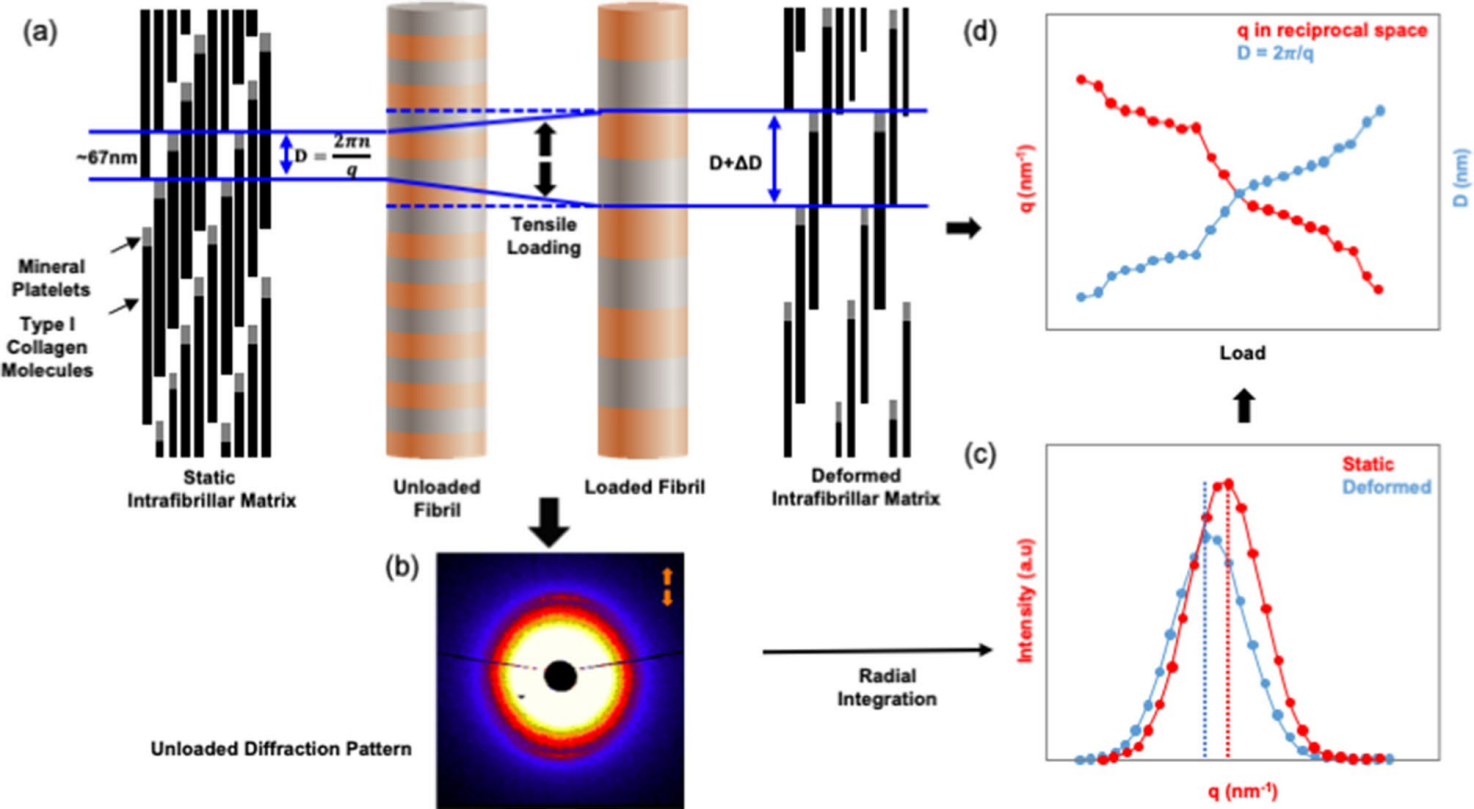
Schematic diagram showing fibril deformation and the SAXD method for measuring fibril strain. (**a**) The 67 nm periodic axial arrangements of type I collagen molecule in the unloaded (i.e. static) and loaded (i.e. deformed) state. D space is the distance between mineral platelets. (**b**) The corresponding SAXD spectra for the unloaded collagen fibrils as captured by the detector (to the human eye the deformed spectra are imperceptibly different). (**c**) Corresponding peaks obtained from radial integration of SAXD spectra shows the shift from the static to deformed state. (**d**) Graph showing how the SAXD diffraction peak shifts after loading and the inverse relationship to the tensile deformation of D-periodic unit in the type I collagen molecule.

Nanostrain measurements take advantage of the repeating nanoscale structure of bone. Mineralised collagen fibrils have a periodic distribution of the mineral that results in a spatial periodicity of 67 nm within the fibrils; this distance referred to as ‘D’ (Fig. 7a). When exposed to X-rays the periodically spaced mineral blocks interact with the beam creating a diffraction pattern (Fig. 7b). Specifically, small-angle X-ray diffraction (SAXD) patterns appear in bone^10^ (Fig. 7). When the bone, and hence the fibril, stretch ‘D’ increases (Fig. 7a) which results in a change in the diffraction patterns causing the diffraction peaks move (which is imperceptible to the human eye). The measured movement of diffraction peaks is inversely proportional to the change in D, that is ΔD (Fig. 7c). Finally, the fibril strain was calculated as = ${\varepsilon }_{fibril}$ΔD/D_unloaded_ (Fig. 7d). The procedure to determine the position and movement of the diffraction peaks is described elsewhere^46,48^. Specifically, we tracked the 3^rd^ order meridional diffraction peak as the 1^st^ order peak was obscured by the high-intensity mineral spectrum. Also, we radially integrated the spectrum from ± 15° around the load direction (0°) thereby isolating and capturing the strains in fibres that are oriented predominantly along the load direction. These 0° fibres will be exposed to the largest strain during loading, thus, will be the first to fail. We adopted this approach as we were particularly interested in the mechanisms that initiate failure and hence the strains in these 0° fibres were of primary importance.

The mineral strain was also obtained by tracking the movement of X-ray diffraction peaks. In this case, the lattice spacing of the apatite mineral produces diffraction patterns allowing the change of the length of the c-axis of the crystalline hydroxyapatite mineral to be tracked using the 002-diffraction peak^49,50^. The mineral strain, ${\varepsilon }_{mineral}$, was calculated as the change in c-axis length due to loading divided by the length of the c-axis when the specimen was unloaded.

The scale of the periodicity of the lattice spacing in the mineral platelets is less than a nanometer, or about 2 orders of magnitude lower than the 67 nm periodicity of the mineralized collagen fibrils. Therefore, the spacing of the diffraction pattern (which is inversely proportional to the scale of the periodicity) spans a wider angle in the mineral case. Hence, for the analysis of mineral strain wide-angle X-ray diffraction (WAXD) set up was required in contrast to the small angle (SAXD) set-up that was used for the analysis of the fibril strain (Fig. 6).

Therefore, the sample-detector distance was 0.175 m and 6.852 m for WAXD and SAXD spectra, respectively (Fig. 6)^46^. The detectors used were the PILATUS 2 M and RAPID, which allowed for fast measurements during the mechanical testing. Samples were scanned in real-time during testing at 2.5-s intervals (X-ray exposure time of 0.5 s). The wavelength was 1 Å with a corresponding X-ray energy of 12.4 keV. Synchrotron X-ray radiation may cause bone damage leading to inaccuracies in measured mechanical behaviour. Barth and colleagues^51^ reported that a radiation dose of less than 30 kGy resulted in minimal bone damage. Hence, the exposure time was set not to exceed 1 s at each scan to minimise radiation dose.

### Statistical analysis

Demographic (age), structural (BV/TV) and mechanical data (critical tissue strain, max tissue strain, strength, peak fibril and mineral strain) were compared between donor groups using nonparametric statistics because Shapiro–Wilk test revealed some of the data were not normally distributed: including Control tissue strain; Fx-Untreated critical strain and fibril strain; Fx-BisTreated tissue and mineral strain. Descriptives were presented as median and interquartile range and compared between donor groups using Kruskal–Wallis and pairwise Mann–Whitney* U* tests.

Tissue-level stresses versus strains curves were plotted for every bone specimen in each of the three groups and showed peak stress (nUTS), Fig. 2a. Similarly, peak strains were apparent when mineral and fibril strain were plotted against tissue strain (Fig. 2b,c). The critical tissue strains at nUTS (${\varepsilon }_{Tissue}^{nUTS}$), max fibril strain (${\varepsilon }_{Tissue}^{\max \; fibril}$) and max mineral strain (${\varepsilon }_{Tissue}^{\max \; mineral}$) were extracted from these curves for each of the 30 the donors. Then the associations between the critical strain (${\varepsilon }_{Tissue}^{nUTS}$) and the nano-level strains of ${\varepsilon }_{Tissue}^{\max\;fibril}$ and ${\varepsilon }_{Tissue}^{\max\;mineral}$ were investigated using paired ANOVA with Tukey’s post hoc.

The correlation between tissue-level properties (i.e. critical tissue strain and strength) and the nanoscale properties (i.e. peak fibril and mineral strain) was investigated using a bivariate linear regression with correlation coefficient *r*^2^. The residuals exhibited normal distributions in QQ plots. All statistical analyses were performed using IBM SPSS Statistics 23 (Armonk, New York) and the graphs were generated with GraphPad Prism 8 (San Diego, California).

## Supplementary information

Supplementary Figure S1.

Supplementary Table S1.
