# Supplementary figures and images for: Nanoscale mechanisms in age-related hip-fractures
Source: Sci Rep. 2020 Aug 26;10:14208. doi: 10.1038/s41598-020-69783-5 (PMC7450077; doi:10.1038/s41598-020-69783-5)

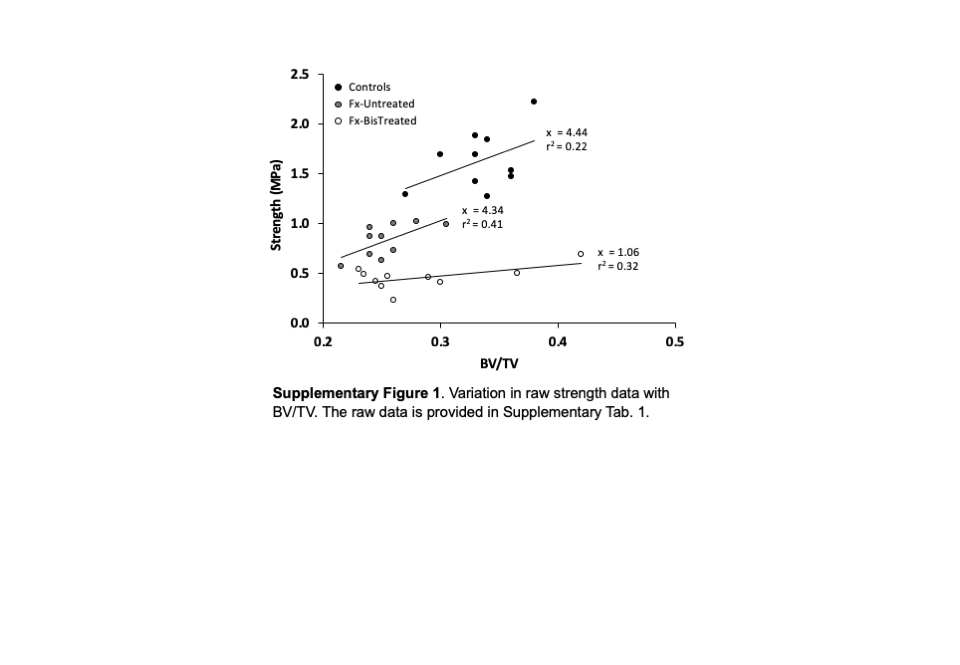

Supplement: Supplementary file 1 — Supplementary Figure S1. [file 41598_2020_69783_MOESM1_ESM.tiff]

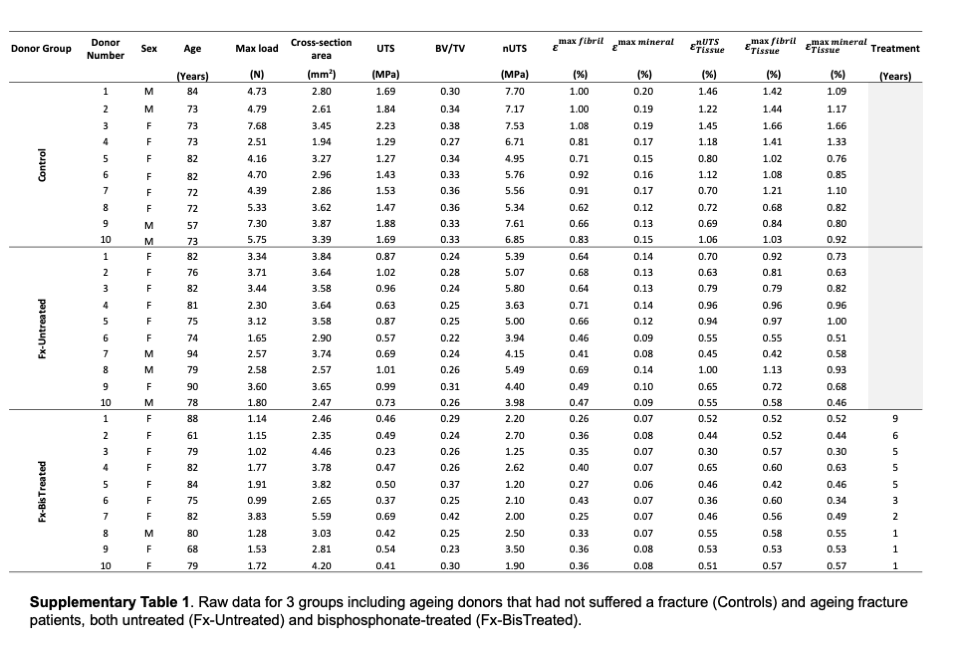

Supplement: Supplementary file 2 — Supplementary Table S1. [file 41598_2020_69783_MOESM2_ESM.tiff]
